# The Institutional Library

**Published:** 1917-02-03

**Authors:** 


### THE INSTITUTIONAL LIBRARY.

### FIRE PREVENTION IN INSTITUTIONS.

A writer of a manual on this important subject is faced by the same difficulty as the experts who ten years ago were asked by the Council of King Edward's Hospital Fund for London to draw up general rules which might be circulated to various hospitals with a view to assisting them to secure a greater degree of protection against fire. Clear, simple, and definite rules may be easily prepared to provide an ideal system of fire protection and fire prevention for a number of institutions which are all constructed on one plan, but in this country, no less than in the United States, institutions housing the sick are constructed on very many different plans, and require many different schemes of fire protection and prevention. The writer of this manual* adequately grasps this obvious truth, and affords us not only general rules, but information relative to what is advisable in a number of varying cases, and as an instance of his exhaustive methods may be quoted a warning against the danger of wood-shingle roofs becoming ignited by means of sparks from chimneys or passing locomotives.

Dr. Eichel and the experts of King Edward's Fund, when we deal with generalities, agree in the following axioms:

(1) Where the building has not been planned with a view to fire risks, it must be amended by added fire-escapes and methods of egress for use in case of danger. (2) Fire-prevention equipment must be both purchased and placed under the guidance of an expert. The equipment and the escapes must be periodically examined, overhauled, and maintained in efficiency. (3) The staff of the institution must be constantly drilled in the use of the equipment, and in the measures to be taken in case of alarm for the safety of the inmates (which comes first), and the safety of the building (which comes second). (4) Co-operation with the local fire department is advisable. (5) The possibilities of the water supply must be clearly understood. These are the headings of the chapters of a manual which must be written under expert guidance, by each institution for itself according to its peculiarities of construction, its position, and the nature of the ailments from which its patients are suffering.

Building upon the framework of these general rules, the following amplifications may be presented for consideration:

(1) If the building contains blind alleys or places where a number of persons may be shut off from escape, these points of danger must be carefully examined and means of escape provided by iron ladders or stairs of a type that provides easy and safe use, or by egress to other parts of the building or to adjacent buildings from which escape is possible. All doors which would be conceivably used in an emergency must be either kept unlocked or, if locked for administrative reasons, have a key in a small glass-fronted cupboard which can be readily broken open. The heating and lighting systems should be examined from the point of view of fire protection, as there are many dangers connected with inferior wiring for electricity, unsuitable gas brackets and stoves, and overheated steam-pipes. No accumulations of rubbish or unnecessary stocks of combustible materials should be allowed. Regulations as to smoking must be rigorously enforced, and the amount of decorations used at Christmas and other festivals reduced to safe limits.

(2) The equipment depends upon the institution. A hospital of, say, fifty beds or upwards will require fire hydrants on each floor, with a sufficiency of hose to reach any part of the area intended to be covered. In addition, there should be buckets of water hanging within easy reach, and a certain number of chemical extinguishers close to each ward, and in other suitable parts, for use in small outbreaks, or while the hydrants are being prepared for working. This auxiliary apparatus should be sufficiently light to be used by women. The whole equipment must be constantly overhauled in order to ascertain that it is in permanent good order; the hose must be tested periodically with full pressure of water. The writer of the manual under review is not in favour of any fire extinguisher employing chemical powder nor of the familiar hand-grenade.

(3) But the best apparatus in the world will be of little use if those who attempt to use it have not been properly instructed, and such instruction must be given very frequently, for a hospital population is constantly changing. However modest the establishment of a hospital, money spent on expert advice and direction is well expended.

But putting out the fire is one thing, and providing for the safety of the inmates another. Necessarily, the functions of the staff in the event of fire will depend upon the character of the institution, but in any case certain persons must understand that it is their main duty to remove and help the patients while others are told off to fight the flames. The number of stretchers available will depend upon the proportion of sick who are quite helpless, and those who remove the patients must remember that they also have to protect them against exposure, and provide accordingly, should the patients have to be taken into the open air.

(4) In large towns most hospitals are on the telephone, and there is a prearranged message in case of a fire outbreak. In addition, the local fire alarm should be operated, and its position having been carefully noted, a particular member of the staff should be entrusted with the duty of making

* *A Manual of Fire Prevention and Fire Protection for Hospitals.* By Otto R. Eichel, M.D. Director, Division of Sanitary Supervisors, New York Department of Health. (New York: John Wiley and Sons. London: Chapman and Hall. 4s. 6d. net.)

use of it. The local fire brigade should be summoned in the case of every outbreak.

(5) The possibilities of the water supply must be noted, and means of increasing the pressure, if they are available, should be clearly understood.

When an outbreak occurs the gas supply must be turned off at the main as quickly as possible, and the electric current disconnected after all inmates are out.

After a fire important prevention work remains. Where steam boilers are in use, which are fed by tank water which has been used in the hydrants, the supply of water to the boilers must be continued, or the fires drawn, otherwise there is danger of an explosion. All dampness in the walls must be noted, as there is a possibility of further risk through the short-circuiting of electric-current wires affected by damp.

When an institution has drawn up its rules, which should be short, simple, and easily remembered, copies should be posted all over the building. As people will often read posted notices which appear to be fresh, these sheets of rules should be replaced from time to time by other sheets of the same rules printed in a different coloured ink. This plan, founded on a knowledge of the psychology of hospital workers, may be found useful in respect of all kinds of displayed notices.

Many of the foregoing are general considerations common to Dr. Eichel's useful manual and to the Report of the King's Fund experts, but, as the former says, "each hospital may present a separate problem." Happily a fire in a hospital, where there are intelligent people about night and day from one year's end to another, is a rare thing; but if an outbreak should occur, coolness on the part of the staff, one result of proper instruction in what must be done, may save a disaster.

---

**The Red Cross in France.** By Granville Barker. With a Preface by Sir Frederick Treves. (London: Hodder and Stoughton. 3s. 6d.)

Those ignorant of the vast scope of the Red Cross Society and the Order of St. John will find enlightenment in Mr. Granville Barker's volume; and if they find its somewhat flippant style unworthy of the great subject it may encourage them to seek more solid information elsewhere. Sir Frederick Treves, in his preface, describes the headquarters of the Society in July 1914 as consisting of a secretary, two clerks, and a boy, working in three small rooms to form Voluntary Aid Detachments "to succour our soldiers in the event of an invasion which was as chimerical as the invasion of the island of Lilliput. The passer-by may have ventured to think these diligent men would be as well employed if they were enlisting recruits for the quest of the Holy Grail." And now, with their ambulances in hundreds, nurses and orderlies in battalions, stores to the value of thousands of pounds a week, and hospitals and rest-stations in England and abroad almost beyond counting, the joint Societies "stand to show that people trying to help are not a rabble of sentimental fools, but a disciplined, capable body of men and women, with their hearts in the job, and brains that are sometimes second to none in the Empire."

**Care and Feeding of Infants and Children: A Text-Book for Trained Nurses.** By W. R. Ramsey, M.D. (London: J. B. Lippincott Company. 9s. net.)

This beautifully illustrated and attractively written volume suffers from the fundamental disadvantage that it is intended for American nurses, and much of its advice on the rearing of children is inapplicable to our climatic conditions and domestic arrangements. We doubt whether the English nurse will often find, for example, mothers who are able and willing to provide shower-baths and entirely separate sleeping apartments for their infants, or doctors who would agree that infantile diarrhœa of every variety is practically always caused by over-feeding. These divergences of outlook may well be forgiven when the outstanding merits of certain other sections are considered. There is a very useful chapter on the distinctive features of the anatomy and physiology of the child, and the information on milk is much fuller and better than in most works of this description. The latter part of the book contains as much pathological and theoretical teaching on the diseases of childhood and infancy as any nurse is likely to want. Dr. Ramsey has a gift of clear and simple writing which always avoids superficiality, and his teaching is thoroughly sound.

**At Suvla Bay.** By John Hargrave. (London: Constable. Pp. 182. Price 5s. net.)

The author of this book served as a sergeant in a field ambulance of the unlucky 10th Division, which was shot to pieces at Suvla Bay in August 1915. Like most other eye-witnesses of what took place, he holds that a born leader could have achieved blinding success instead of ghastly failure on that occasion. The observations of so acute a critic as Sergeant Hargrave are valuable; and many of his vivid pen pictures, both in words and in line, are highly successful. His book suffers, however, from an excess of self-consciousness, a too evident striving for effect. It is distinctly worth reading, but we doubt whether it will really live among the vast literature of which this war is certain to be the centre.

**Practical Text-Book of Midwifery for Nurses.** By Robert Jardine, M.D., etc., Professor of Midwifery in St. Mungo's College, Glasgow. (London: H. Kimpton, 263 High Holborn, W.C. 5s. net.)

The preface and introduction to this well-known manual deal with the effect of the Scottish Midwives Act on maternity nurses in Scotland, for whom the book was originally written. An excellent chapter on Infant Feeding, by Dr. Leonard Findlay, has been added. It is interesting to note the differences in nomenclature and practice which obtain in North Britain. How many English nurses would recognise "hives" as a generic term among the Scottish poor for various childish complaints, including rashes and diarrhœa? "Labour tea," we are told, is made by pouring boiling water over dry ergot, but it is more than doubtful if an English chemist would supply the powder for that purpose. In many ways the book has been brought up to date, so it is a little surprising that no mention has been made of scopolamine-morphine treatment, which has been used in Scotland far more than in England. In the chapter on Contraction of the Pelvis the author assumes that midwives do not carry callipers; he would probably be surprised to find how many English midwives carry and use them to good purpose. The illustrations are for the most part extremely helpful, the sections well arranged, and the explanations throughout admirably expressed.

